# Cinnamomum-Longepaniculatum-Leaves-Based Fe-N Doped Porous Carbon as an Effective Oxygen Reduction Catalyst

**DOI:** 10.3390/molecules30081708

**Published:** 2025-04-10

**Authors:** Yashu Li, Nan Wang, Lu Zhao, Xuanhe Liu, Lin Wang, Chengcheng Xie, Jing Li

**Affiliations:** 1 Engineering Research Center of Ministry of Education for Geological Carbon Storage and Low Carbon Utilization of Resources, Beijing Key Laboratory of Materials Utilization of Nonmetallic Minerals and Solid Wastes, National Laboratory of Mineral Materials, School of Materials Science and Technology, China University of Geosciences, Beijing 100083, China; liyashu1832@163.com (Y.L.); wangnan7122@163.com (N.W.); lzhao@cugb.edu.cn (L.Z.); liuxh@cugb.edu.cn (X.L.); 2 School of Humanities and Tourism, Yibin Vocational and Technical College, Yibin 644100, China; 3 Key Laboratory of Bio-Inspired Smart Interfacial Science and Technology of Ministry of Education, School of Chemistry, Beihang University, Beijing 100191, China; chmlij@buaa.edu.cn

**Keywords:** biomass carbon, heteroatom doping, iron phthalocyanine, electrocatalysis, oxygen reduction reaction

## Abstract

Developing low-cost, efficient, and scalable non-precious metal electrocatalysts for the oxygen reduction reaction (ORR) remains a critical challenge in the field of energy conversion. Among various candidates, Fe-N-doped carbon materials have garnered attention as promising alternatives to commercial Pt/C catalysts for ORR. In this study, we report an Fe-N catalyst synthesized by incorporating iron phthalocyanine with *Cinnamomum longepaniculatum* waste leaves as the carbon source. This catalyst exhibited an excellent four-electron ORR activity and the half-wave potential (E_1/2_) reaches 0.875 V, which was superior to that of commercial Pt/C (E_1/2_ = 0.864 V). Additionally, the catalyst exhibits superior methanol tolerance and stability compared to commercial Pt/C. This approach, which utilizes biomass waste for the synthesis of electrocatalysts, not only provides an effective solution for reducing environmental waste but also addresses the issue of sluggish cathodic ORR kinetics in fuel cells, making it suitable for low-cost, large-scale industrial production.

## 1. Introduction

Oxygen reduction reaction (ORR) is a critical electrochemical process occurring at the cathode of fuel cells; however, its inherently slow kinetics significantly hinder the performance of these energy devices [1]. Thus, developing and refining novel electrocatalysts for the ORR process has become a pivotal focus for global academic and industrial efforts. While Pt/C remains the benchmark electrocatalyst for ORR, its limited abundance and high cost present substantial challenges for large-scale applications [2,3]. In response, non-precious metal-based carbon materials have emerged as promising alternatives, owing to their advantages of affordability, high catalytic activity, and long-term stability [4]. Metal–nitrogen co-doped carbon catalysts (M/N-C) have been shown to generate more active sites and modulate the electronic structure of the catalyst [5,6] and possess higher ORR activity and stability, which have been proven to be catalytic alternatives that can replace platinum carbon catalysts (Pt/C) [7,8]. However, most of the raw materials used in the preparation of traditional metal–nitrogen double-doped carbon catalysts are metal salts (such as chlorides, nitrates, etc.), which probably lead to the aggregation of metal atoms in the prepared catalysts [9], thereby slowing down the kinetics of the oxygen reduction reaction.

Metal phthalocyanines are a well-established class of organic complexes characterized by a highly conjugated macrocyclic structure with a central chelated metal ion. Using phthalocyanine as a metal dopant can ensure the dispersion of metal ions in the prepared catalyst and the stability of the oxygen reduction electrocatalyst [10,11]. In recent years, phthalocyanine-doped carbon materials, such as carbon spheres [12], carbon nanotubes, and graphene [13], prepared through top–down approaches have been widely used for electrocatalytic oxygen reduction. By preparing iron phthalocyanine-modified derivative carbon materials, Praat et al. [14] explored that more carbon nanotubes structures can increase the overall mesoporosity of the catalyst, so that the ORR activity is higher, which exhibits an onset potential and a half-wave potential of 0.93 V and 0.77 V, respectively. Kumar et al. [13] synthesized catalysts with carbon nanotubes and mesoporous carbon as the carrier have FeNi nanoparticles of about 5–10 nm. This small-size nanoparticle structure can promote rapid electron transfer between carbon matrix and nanoparticle, thus achieving an efficient ORR performance [15,16]. In addition, metal–nitrogen centers and metal oxides are also considered to be the active sites of ORR. However, the carbon supports typically used in phthalocyanine-doped materials are derived from relatively expensive carbon nanomaterials, and their complex synthesis procedures, coupled with often unsatisfactory catalytic performance, limit their scalability for industrial applications.

Biomass waste is increasingly utilized in catalysis due to its cost effectiveness, environmental sustainability, and low-cost nature. It serves as both an efficient catalyst and a carbon support, such as rice straw [4] and fruit peel [17], being activated and pyrolyzed to exhibit high surface areas and porosity [18]. These materials can then support macrocyclic metal complexes like phthalocyanine or porphyrin, resulting in catalysts with excellent oxygen reduction reaction (ORR) performance [19]. In this study, *Cinnamomum longepaniculatum* (*C. longepaniculatum*) waste leaves, which are rich in cellulose, hemicellulose, and lignin [20], are used as carbon precursors. The leaves are pyrolyzed with KHCO₃ and melamine as activators and nitrogen dopants, producing nitrogen-doped porous carbon (NBC). Iron phthalocyanine (FePc) is then loaded onto NBC to form Fe-N doped porous carbon (Fe-NBC), both of which demonstrate high ORR activity. The large surface area, abundant porosity, and increased nitrogen content (7.25% for NBC and 13.63% for Fe-NBC) enhance their ORR performance, with onset and half-wave potentials comparable to those of commercial Pt/C. This method offers a cost-effective approach to developing high-performance ORR electrocatalysts with potential for industrial-scale applications.

## 2. Results and Discussion

### 2.1. Morphology and Structure Characterization

**Scheme 1:**
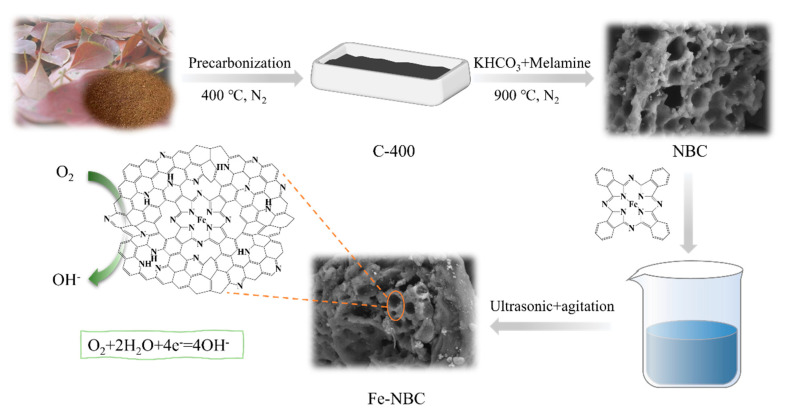
Synthesis schematic of biomass-based porous carbon.

**Figure 1:**
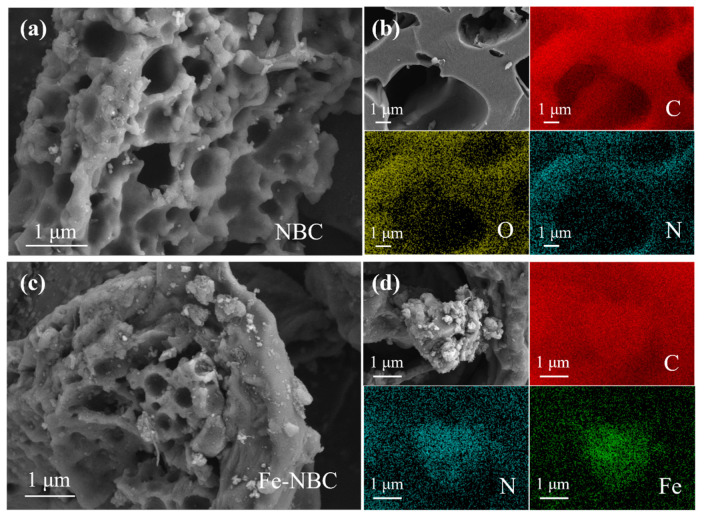
(**a**) SEM image of NBC and (**b**) the corresponding element mapping diagram; (**c**) the SEM images of Fe-NBC and (**d**) the corresponding element mappings.

The method for preparing biomass-based porous carbon from waste leaves of *C. longepaniculatum* is introduced by Scheme 1. First, the waste leaves are pre-carbonized to remove the crystalline water within them. Subsequently, they are uniformly ground with an activator and nitrogen dopant, followed by pyrolysis under a nitrogen atmosphere to prepare nitrogen-doped porous carbon (NBC). And then FePc is loaded to obtain Fe and N co-doped porous carbon (Fe-NBC). The resulting nitrogen-doped biochar and Fe and N co-doped biochar were characterized using SEM. As shown in Figure 1a, the surface of the NBC catalyst exhibits a distinctly rough pore structure. After doping Fe on the biochar surface (Figure 1c), Fe-NBC also contains a relatively rich pore structure, with a uniform distribution of Fe element observable. This pore structure facilitates electron transfer and the exposure of active sites [4]. The observed pores might be attributed to the presence of the activator KHCO_3_ and the dopant melamine, which promote the carbonization process. In comparison to the biochar (C) produced without the activator and dopant shown in Figure S1, the material exhibited larger, more irregular pores, with nanoparticles randomly aggregated and a notably rough surface texture. Additionally, elemental mapping characterization of both types of catalysts (Figure 1b,d) indicates that these elements are evenly distributed within the catalyst, with no significant aggregation of elements observed.

**Figure 2:**
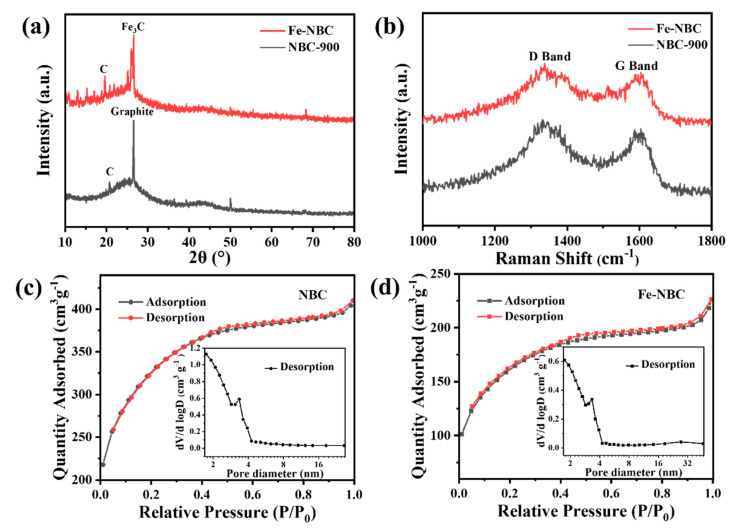
(**a**) XRD patterns and (**b**) Raman spectrum of NBC and Fe-NBC; N_2_ adsorption−desorption isotherms and pore size distribution of (**c**) NBC and (**d**) Fe−NBC.

Figure 2a shows the X-Ray powder diffraction (XRD) patterns of NBC and Fe-NBC, which were used to determine the composition and crystalline phases of the biochar derived from waste leaves. For NBC, the peak at 26.6° corresponds to graphite carbon [21], indicating that the crystallinity of the prepared NBC is good, while the diffraction peak at 20.7° corresponds to amorphous carbon [22,23]. After the incorporation of Fe, the peak at 26.4° corresponds to Fe_3_C (PDF 85-0871) and, compared to NBC, the intensity of the graphite carbon diffraction peak decreases, with a slight negative shift observed. This results in a widening of the interlayer spacing, making it easier to be doped with Fe [24]. The microstructure of the prepared catalyst was investigated by Raman spectroscopy, as shown in Figure 2b. The peaks at 1350 cm^−1^ and 1590 cm^−1^ are attributed to the D and G bands, respectively. The D band represents sp^3^ hybridized carbon atoms, which are associated with defect states in the carbon materials, while the G band is related to in-plane vibration of sp^2^ carbon atoms and typically indicates the crystallinity of the carbon [25,26]. The intensity ratio of the D and G bands (I_D_/I_G_) reflects the degree of disorder in the carbon structure. The ID/IG ratios of the NBC and Fe-NBC catalysts are 1.09 and 1.04, respectively, indicating a decrease in the ratio of the D and G bands (I_D_/I_G_) after doping with Fe, suggesting that the graphitization of the catalyst increases following transition metal doping [27].

The specific surface area and pore structure of the catalysts were characterized and analyzed by BET. The N_2_ adsorption–desorption isotherms and pore size distribution for NBC and Fe-NBC are shown in Figure 2c and 2d, respectively. Table S1 presents the pore structure parameters of the catalysts prepared based on different ratios of the two catalysts. The results indicate that among the NBC prepared at different ratios, the NBC-1:2:2 (NBC) catalyst has the largest specific surface area of 1163.6 m^2^/g, with a pore distribution mainly in the range of 1–4 nm, which belongs to the category micropores and mesopores. The specific surface area of the prepared Fe-NBC is 580.5 m^2^/g, with its pore distribution primarily also in the 1–4 nm range. These pore structures are conducive to the exposure of active sites and the mass transfer during the ORR process [4]. The N_2_ adsorption–desorption curves of the two catalysts exhibit similar shapes, both showing Type IV isotherms with H3-type hysteresis loops, indicating the presence of substantial mesopores in the catalysts. However, in the P/P_0_ range of 0.95–1.0, the curve becomes noticeably steeper after the introduction of Fe, suggesting that the catalyst contains larger pores [28]. After the incorporation of Fe, the specific surface area of the catalyst significantly decreased, and the average pore size increased from 2.53 nm to 3.03 nm. This may be because the incorporation of Fe destroyed the pore structure of the catalyst and made the pores larger [29], resulting in the change in the specific surface area and pore size of the catalyst.

**Figure 3:**
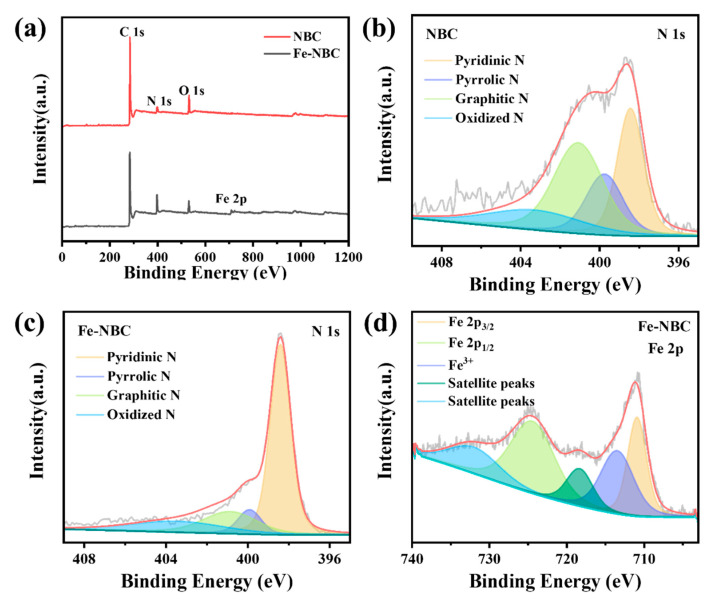
(**a**) XPS full spectra of NBC and Fe-NBC; (**b**) N 1s fine spectrum of NBC; (**c**) N 1s fine spectra; and (**d**) Fe 2p fine spectra of Fe-NBC.

The electronic structure of the catalysts was characterized by X-Ray photoelectron spectroscopy (XPS). Figure 3a shows that both NBC and Fe-NBC exhibit the presence of carbon (C), nitrogen (N), and oxygen (O) at binding energies of 285 eV, 400 eV, and 534 eV, respectively, while the presence of iron (Fe) is observed at 707 eV in Fe-NBC. The N 1s fine spectrum of both catalysts can be divided into four peaks corresponding to pyridinic N, pyrrolic N, graphitic N, and oxidized N [30]. These peaks in NBC are located at 398.4 eV, 399.7 eV, 401.1 eV, and 403.1 eV, while in Fe-NBC, they are at 398.4 eV, 399.9 eV, 400.9 eV, and 403.2 eV, respectively. Pyridinic N and graphitic N are recognized as active sites in various nitrogen configurations [31,32]. Table S2 shows that the content of pyridine N and graphite N in the two catalysts is relatively high, which is beneficial for the ORR process. In the Fe 2p fine spectrum of Fe-NBC, peaks at 710.9 eV and 724.4 eV are attributed to Fe 2p_3/2_ and Fe 2p_1/2_, respectively; the peak at 713.4 eV corresponds to Fe^3+^, and the peaks at 718.3 eV and 731.9 eV are satellite peaks [33], indicating successful incorporation of Fe. As shown in Table S3, the content of N in NBC is 7.25%, while in Fe-NBC, it increases to 13.63%. This increase may be due to the addition of FePc, which elevates the atom content of N and provides more active sites [34], thereby enhancing the ORR activity of the catalyst.

### 2.2. Electrochemical Performance

The different catalysts were evaluated for their electrocatalytic oxygen reduction reaction (ORR) performance under O_2_ saturation by a rotating disk electrode (RDE). The linear sweep voltammetry (LSV) curves were tested under O_2_-saturated conditions in a 0.1 M KOH electrolyte. As shown in Figure S2a, in the absence of Fe doping, the NBC prepared with a mass ratio of carbon precursor, N dopant, and activator of 1:2:2 exhibited the best electrocatalytic ORR performance. It shows an onset potential (E_onset_) and half-wave potential (E_1/2_) reaching 0.95 V and 0.84 V, respectively, comparable to commercial Pt/C (E_onset_ = 0.96 V; E_1/2_ = 0.864 V). This can be attributed to the large specific surface area and abundant pore structures of NBC, as well as the doping of N, which exposes and increases more active sites [1]. The activity of the catalyst was further confirmed from the perspective of reaction kinetics, as a smaller Tafel slope indicates a faster electron transfer rate during the ORR process [35]. The Tafel slope for NBC-1:2:2 (NBC) was found to be the lowest at 76.10 mV/dec, indicating that its mass transfer was relatively fast (Figure S2b) and that good ORR activity was observed. As shown in Figure S2c, the cyclic voltammetry (CV) curves of the best-performing sample were also plotted, showing distinct differences under N_2_ and O_2_ saturation conditions. A notable reduction peak (0.806 V) was observed under O_2_ saturation, while no significant peak appeared under N_2_ saturation, further indicating the electrocatalytic ORR activity of the prepared catalyst. As shown in Figure S2d, testing with a rotating ring–disk electrode (RRDE) indicated that the H_2_O_2_ yield of the catalyst is below 5%, suggesting minimal byproduct formation during the catalytic process. The electron transfer number (n) was around four, indicating that the ORR process of the catalyst was dominated by the four-electron transfer pathway [36].

Methanol tolerance is crucial for the practical application of fuel cells, making it an important test method to evaluate the anti-toxic properties of catalysts [37]. As shown in Figure S2e, at a potential of 0.7 V (vs. RHE), the addition of 5 mL of methanol around 400 s did not result in a significant decrease in the current density of the catalyst. In contrast, the current density of commercial Pt/C decreased significantly due to the oxidation of MeOH on the electrode surface, indicating that this catalyst exhibits high methanol resistance. In practical applications, the life of the catalyst is essential, and durability testing is therefore indispensable. In the polarization range of 0.6–1.0 V, the catalyst underwent 5000 CV cycles before testing the LSV curves. The results (Figure S2f) show that after cycling, the half-wave potential (E_1/2_) of NBC-1:2:2 (NBC) shifted negatively by 4 mV, demonstrating high stability.

**Figure 4:**
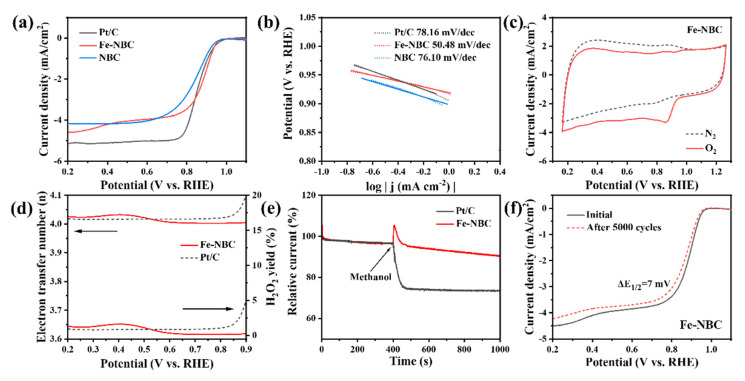
(**a**) LSV curves and (**b**) Tafel slopes of Fe−NBC and NBC; (**c**) CV curves of Fe−NBC at N_2_ and O_2_ saturation; (**d**) H_2_O_2_ yield and electron transfer number and (**e**) methanol tolerance of Fe−NBC and Pt/C; (**f**) LSV curves of Fe−NBC catalysts before and after 5000 cycles.

Similarly, the electrocatalytic ORR performance of Fe-NBC was tested. From the LSV curve (Figure S3a), the ORR activity is highest when the mass ratio of FePc to NBC is 1:2, with an onset potential (E_onset_) and half-wave potential (E_1/2_) reaching 0.964 V and 0.875 V, respectively, outperforming NBC catalysts (Figure 4a) and commercial Pt/C (E_1/2_ = 0.864 V). Table 1 shows the comparison of ORR activity between this work and recently published Pt-free catalysts, indicating that the prepared Fe-NBC has relatively excellent ORR performance. Figure 4b illustrates the Tafel slopes of different ratios of Fe-NBCs. Fe-NBC (1:2) shows the smallest Tafel slope of 50.48 mV/dec, lower than that of commercial Pt/C (78.16 mV/dec) (Figure S3b), indicating a faster electron transfer rate and higher ORR activity [35]. The CV curves tested under O_2_ saturation show an oxygen reduction peak at 0.863 V (Figure 4c), which is higher than that of the NBC catalyst (0.806 V) and commercial Pt/C (0.825 V). The higher oxygen reduction peak potential represents higher ORR activity [3]. The addition of Fe increases the average pore size of the catalyst, facilitating improved mass transfer [38]. Furthermore, the incorporation of FePc elevates the nitrogen content from 7.25% to 13.63%, potentially providing more active sites. Notably, the Fe-N_4_ structures in iron phthalocyanine are considered key active sites with higher catalytic activity in the ORR process [23].

As shown in Figure 4d and Figure S4, the test of the rotating ring–disk electrode (RRDE) indicated that the H_2_O_2_ yield of the catalyst is below 2.5%, which is lower than that of the NBC catalyst and Pt/C. This suggests that there are fewer byproducts in the catalytic process. The electron transfer number (n) is around four, indicating that the ORR process primarily follows a four-electron transfer pathway. The methanol tolerance and durability of Fe-NBC were also tested (Figure 4e,f). After adding methanol, there was no significant change in the current density of the catalyst, demonstrating good resistance to methanol. The LSV curves before and after 5000 cycles further confirmed its high stability (E_1/2_ negatively shifted by 7 mV). The long-term stability tests of the catalysts under oxygen-saturated conditions (Figure S5) revealed that NBC and Fe-NBC catalysts retained 84% and 90% of the initial current density after 20,000 s of continuous operation, while the current density of Pt/C decreased to 66% of the initial current density after reaction, indicating the superior long-term stability of the NBC and Fe-NBC catalysts. These results indicate that Fe-NBC has high electrocatalytic ORR activity.

## 3. Materials and Methods

### 3.1. Materials

The waste *C. longepaniculatum* leaves are obtained after extracting camphor oil from *C. longepaniculatum* leaves. They are provided by Yibin City, Sichuan Province. KHCO_3_ is purchased from Shandong Keyuan Biochemical Co., Ltd., Jinan, China. Melamine is purchased from Sinopharm Chemical Reagent Co., Ltd., Shanghai, China. FePc (90%) is purchased from Shanghai Aladdin Biochemical Technology Co., Ltd., Shanghai, China. KOH is purchased from Beijing Yili Fine Chemical Co., Ltd., Beijing, China. HCl is purchased from Jingchun Reagent Company, Shanghai, China. Nafion (5 wt.%) is purchased from Jieshikai Biotechnology Co., Ltd., Shanghai, China. Pt/C (20 wt%) is provided by Beijing Innochem Technology Co., Ltd., Beijing, China. The chemical reagents used in this study are of analytical grade without any processing.

### 3.2. Preparation of Catalysts

Pre-carbonization: The washed waste leaves of *C. longepaniculatum* were dried in an oven, ground into powder by a vibration mill and passed through a 1.5 mm sieve. An appropriate amount of waste leaf powder of *C. longepaniculatum* was weighed and heated to 400 °C at a rate of 5 °C/min in a tube furnace in N_2_ atmosphere for 2 h. After cooling to room temperature, the carbon precursor C-400 was obtained.

Preparation of N-doped biochar catalyst: The carbon precursor, activator KHCO_3_, and N-doped melamine were mixed at a mass ratio of 1:1:1, 1:2:2, and 1:3:3 and ground evenly. The mixture was heated to 900 °C at a rate of 5 °C/min in a tube furnace under N_2_ atmosphere and held for 2 h. After cooling to room temperature, it was stirred with 0.5 M HCl for 2 h, washed to neutral, and dried to obtain N-doped biochar catalysts NBC-1:1:1, NBC-1:2:2 (NBC), and NBC-1:3:3.

Preparation of Fe, N-doped biochar catalyst: FePc and prepared NBC-1:2:2 were ultrasonicated in DMF at a mass ratio of 1:1, 1:2, 1:3, 2:1 for 1 h, then stirred for 5 h, washed with ethanol and dried to obtain Fe, N-doped biochar catalysts Fe-NBC-1:1, Fe-NBC-1:2 (Fe-NBC), Fe-NBC-1:3, Fe-NBC-2:1.

### 3.3. Physicochemical Characterization

The surface morphology of the catalyst was observed by a German ZEISS Gemini SEM 300 scanning electron microscope (SEM, Oberkochen, Germany). The crystal structure of the catalyst was characterized by a D8 Advance X-Ray diffractometer (XRD, Billerica, Germany). Raman spectra were measured using a LabRAM HR Evolution Raman spectrometer (Paris, France). The specific surface area and pore structure were determined by a Micromeritics ASAP 2460 (Norcross, GA, USA) automatic specific surface area and porosity analyzer (BET). The elemental composition and chemical state of the catalyst were characterized by a Thermo Scientific K-Alpha X-Ray photoelectron spectrometer (XPS, Waltham, MA, USA).

### 3.4. Electrochemical Characterization

All electrochemical tests were conducted at the electrochemical workstation of Shanghai Chenhua by the three-electrode method. The counter electrode was platinum wire, the reference electrode was Ag/AgCl electrode, and the electrolyte was 0.1 M KOH solution. The rotating disk electrode coated with biochar catalyst was used as the working electrode (with a loading of 0.28 mg cm^2^). The catalyst ink was prepared by adding 2 mg catalyst to 760 μL ethanol and 40 μL 0.5 wt.% Nafion solution. Cyclic voltammetry curves and linear sweep voltammetry curves were tested at scan rates of 50 and 10 mV/s, respectively.

All potentials were converted to reversible hydrogen electrode (RHE):

(1) E(vs.RHE)=E(vs.Ag/AgCl)+0.197+0.059pH

The H_2_O_2_ yield and the number of transferred electrons n of the catalyst were tested and calculated by the rotating ring–disk electrode (RRDE):

(2) $$n=4\text{×}\frac{I_{d}}{I_{d}{+I}_{r}/N}$$

(3) $$H_{2}O_{2}\%=200\;\text{×}\;\frac{I_{r}/N}{I_{d}{+I}_{r}/N}$$

Among them, I_d_ is the rotating disk’s current density; I_r_ is the rotating ring’s current density; n is the collection efficiency of the Pt ring (0.37).

## 4. Conclusions

In summary, the preparation of ORR electrocatalysts from biomass waste leaves of *C. longepaniculatum* is an economical, environmentally friendly, and sustainable method. N-doped porous carbon (NBC) and Fe, N-doped porous carbon (Fe-NBC) were prepared by a simple pyrolysis method using biomass waste leaves as raw materials. Both types of porous carbon can be used as ORR electrocatalysts and exhibit high ORR activity. The large specific surface area and high nitrogen content are the main reasons for their enhanced catalytic performance. The half-wave potentials of NBC and Fe-NBC are 0.84 V and 0.875 V, respectively. The latter is better than that of commercial Pt/C (E_1/2_ = 0.864 V), demonstrating a superior electrocatalytic oxygen reduction performance. This study confirms a simple and effective strategy for converting biomass waste into ORR electrocatalysts, providing directions for further optimization and potential expansion of this class of catalysts.

## Figures and Tables

**Table 1:** Comparison of ORR performance of electrocatalysts.

C Source	Doping Element	Catalyst	E_onset_	E_1/2_	Ref
			(V vs. RHE)	(V vs. RHE)	
Waste leaves of *C. longepaniculatum* trees	N	NBC	0.95	0.84	This work
Waste leaves of *C. longepaniculatum* trees	Fe, N	Fe-NBC	0.965	0.875	This work
Lignin	Fe, Mn, N	Fe_5_C_2_/Mn, N, S-CNTs	1.04	0.85	[39]
Rice husk	Fe, Si	Fe-N-SiC	0.96	0.87	[40]
Potatoes	Fe, Co, N	Po-FeCo-N-C	-	0.842	[41]
Passion fruit peels	Fe, N	Fe-NPC	0.967	0.872	[38]
Tea leaves waste	Fe, N	Fe-N/C	0.93	0.84	[42]
Yeast	Co, N	CoNC	-	0.854	[43]
Corn stalks	Fe, N	Fe_5_-PANI/C-MCS	1.09	0.85	[44]
Shaddock peel	Fe, N	SP-N_1_-Z_0_._6_/F_0_._5_	0.925	0.828	[45]
Water hyacinth root	Fe, Al, Zn, N	WHR700	0.94	0.78	[46]
Egg gel	Co, N, S	Co-NSEC	0.974	0.842	[47]

## Data Availability

Data are contained within this article and the Supplementary Materials.

## Supplementary Materials

The following supporting information can be downloaded at: https://www.mdpi.com/article/10.3390/molecules30081708/s1. Figure S1. SEM image of biochar. Table S1 Specific surface area and pore parameters of catalysts. Table S2 Percentages of N in different configurations. Table S3 Element content of catalyst determined by XPS. Figure S2. (a) LSV curve and (b) Tafel slope of NBC prepared under different conditions; (c) CV curves of NBC at N_2_ and O_2_ saturation; (d) H_2_O_2_ yield and electron transfer number and (e) methanol tolerance of NBC and Pt/C; (f) LSV curve of NBC catalyst before and after 5000 cycles. Figure S3. (a) LSV curve and (b) Tafel slope of Fe-NBC prepared under different proportions. Figure S4 (a) Ring and disk current densities of Fe-NBC and Pt/C catalysts in RRDE measurements; (b) Partial enlarged detail of ring current densities. Figure S5 The long-term stability tests of Fe-NBC, NBC and Pt/C.
