# Supplementary material for: Cinnamomum-Longepaniculatum-Leaves-Based Fe-N Doped Porous Carbon as an Effective Oxygen Reduction Catalyst
Source: Molecules. 2025 Apr 10;30(8):1708. doi: 10.3390/molecules30081708 (PMC12029488; doi:10.3390/molecules30081708)
Supplement: Supplementary file 1 [file molecules-30-01708-s001.zip › molecules-3567970-supplementary.pdf]

Supporting Information

Cinnamomum-Longepaniculatum-Leaves-Based Fe-N Doped Porous Carbon as an Effective Oxygen Reduction Catalyst

Yashu Li [1], Nan Wang [1], Lu Zhao [1], Xuanhe Liu [1], Lin Wang [1,*], Chengcheng Xie [2,*] and Jing Li [3]

[1] Engineering Research Center of Ministry of Education for Geological Carbon Storage and Low Carbon Utilization of Resources, Beijing Key Laboratory of Materials Utilization of Nonmetallic Minerals and Solid Wastes, National Laboratory of Mineral Materials, School of Materials Science and Technology, China University of Geosciences, Beijing 100083, China; liyashu1832@163.com (Y.L.); wangnan7122@163.com (N.W.); lzhao@cugb.edu.cn (L.Z.); liuxh@cugb.edu.cn (X.L.)

[2] School of Humanities and Tourism, Yibin Vocational and Technical College, Yibin 644100, China

[3] Key Laboratory of Bio-Inspired Smart Interfacial Science and Technology of Ministry of Education, School of Chemistry, Beihang University, Beijing 100191, China; chmlij@buaa.edu.cn

* Correspondence: wanglin712@cugb.edu.cn (L.W.); xiechengcheng@stu.sicau.edu.cn (C.X.)

Supplementary Results

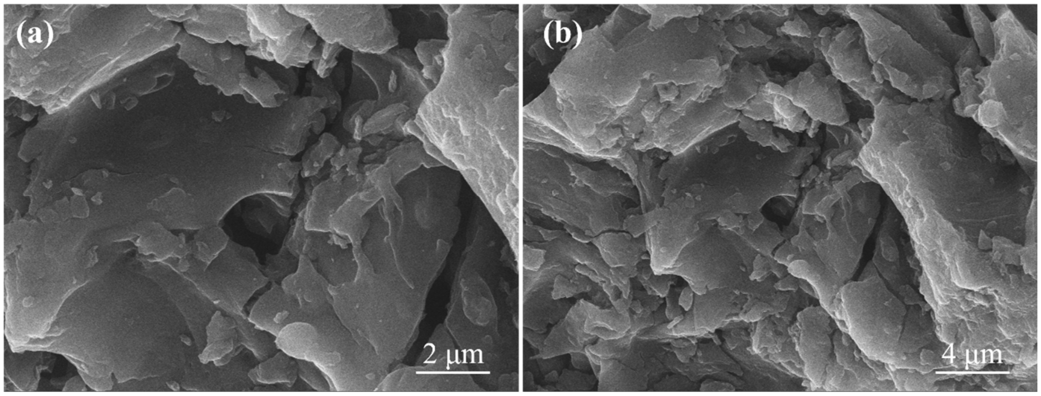

Figure S1. SEM image of biochar.

Table S1. Specific surface area and pore parameters of catalysts.

Catalysts	Surface area (m^2g^{-1})	Pore volume (cm^3g^{-1})	Pore size (nm)
NBC-1:1:1	1086.9	0.5213	2.7539
NBC-1:2:2 (NBC)	1163.6	0.6343	2.5295
NBC-1:3:3	1067.3	0.6622	2.6145
Fe-NBC	580.52	0.3501	3.0284

Table S2. Percentages of N in different configurations.

Catalysts (at%)	Pyridinic N	Pyrrolic N	Graphitic N	Oxidized N
NBC	28.32	18.78	37.22	15.68
Fe-NBC	57.22	7.78	18.61	16.40

Table S3. Element content of catalyst determined by XPS.

Catalysts (at%)	C	O	N	Fe
NBC	83.58	9.17	7.25	—
Fe-NBC	78.63	6.48	13.63	1.26

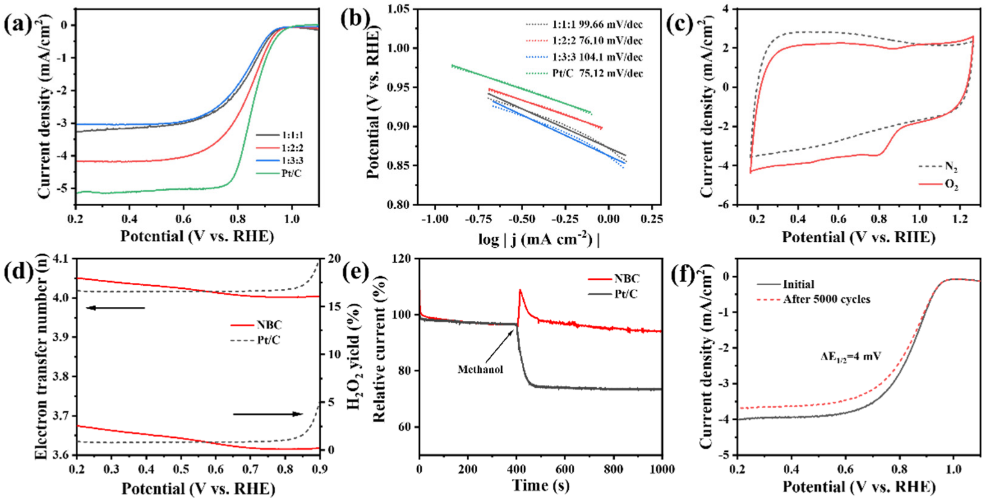

Figure S2. (a) LSV curve and (b) Tafel slope of NBC prepared under different conditions; (c) CV curves of NBC at N_2 and O_2 saturation; (d) H_2O_2 yield and electron transfer number and (e) methanol tolerance of NBC and Pt/C; (f) LSV curve of NBC catalyst before and after 5000 cycles.

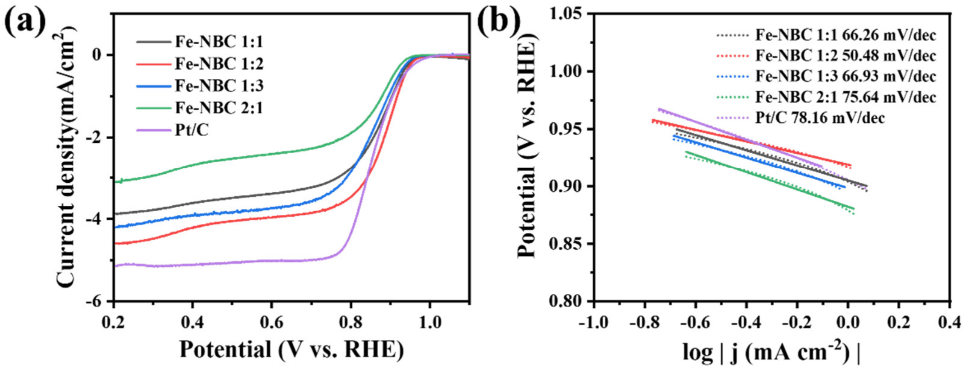

Figure S3. (a) LSV curve and (b) Tafel slope of Fe-NBC prepared under different proportions.

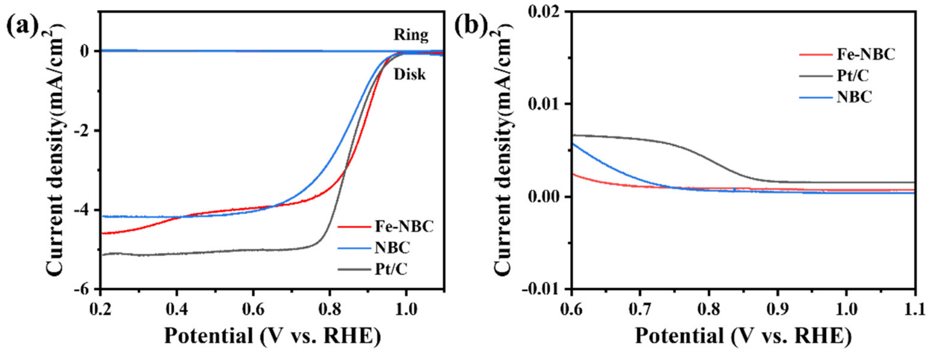

Figure S4. (a) Ring and disk current densities of Fe-NBC and Pt/C catalysts in RRDE measurements; (b) Partial enlarged detail of ring current densities.

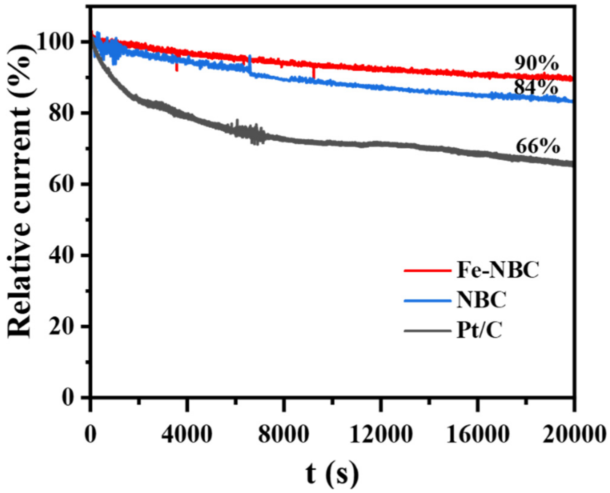

Figure S5. The long-term stability tests of Fe-NBC, NBC and Pt/C.
